# QKI-6 Suppresses Cell Proliferation, Migration, and EMT in Non-Small Cell Lung Cancer

**DOI:** 10.3389/fonc.2022.897553

**Published:** 2022-05-05

**Authors:** Haihua Zhang, Junqiang Li, Feng Tian, Xuan Su, Xinxin Wang, Di Tang, Lei Zhang, Tao Zhang, Yunfeng Ni

**Affiliations:** ^1^ Department of Thoracic Surgery, Tangdu Hospital, Fourth Military Medical University, Xi’an, China; ^2^ Department of Oncology, Tangdu Hospital, Fourth Military Medical University, Xi’an, China; ^3^ Department of Pulmonary and Critical Care Medicine, Tangdu Hospital, Fourth Military Medical University, Xi’an, China; ^4^ Seventh Battalion, Second Cadet Regiment, Fourth Military Medical University, Xi’an, China; ^5^ Department of Oncology, First Affiliated Hospital of Xi’an Jiaotong University, Xi’an, China

**Keywords:** AGR2, non-small cell lung cancer, label-free quantification, tumor progression, QKI-6

## Abstract

The RNA-binding protein quaking homolog 6 (QKI-6) is a tumor-suppressor gene in several cancers. However, its role in non-small cell lung cancer (NSCLC) is unclear. In this study, we aimed to determine the association between QKI-6 expression and survival and clinicopathological features in patients with NSCLC and identify the related mechanisms. Western blot and immunohistochemistry (IHC) were used to detect QKI-6 expression in NSCLC. The effect of QKI-6 on NSCLC cells was determined by overexpression and knockdown assays, and label-free quantitative proteomics and Western blot were used to identify the underlying mechanisms. Low QKI-6 expression level was positively correlated with poor overall survival in patients with NSCLC. Furthermore, QKI-6 overexpression inhibited NSCLC cell proliferation and migration and induced a block in the G0/G1 phase, and QKI-6 downregulation increased proliferation and migration. QKI-6 inhibited EMT processes *via* EGFR/SRC/STAT3 signaling by upregulating AGR2. In conclusion, QKI-6 could be used to develop novel strategies for the treatment of NSCLC.

## Introduction

Lung cancer is the leading cause of cancer-related death worldwide and has a mortality rate of 18.4% (1). Non-small cell lung cancer (NSCLC) comprises approximately 85% of the total cases of lung cancer worldwide (2). Current treatment strategies for NSCLC include surgical resection, targeted therapies, and radiochemotherapy; however, the 5-year overall survival (OS) of patients with NSCLC remains low (15%) (3). Identification of the molecular mechanisms underlying NSCLC progression and, subsequently, the novel therapeutic approaches for NSCLC is therefore critical. Epithelial–mesenchymal transition (EMT), characterized by the conversion of epithelial cells to mesenchymal cell phenotypes, is essential to tumor progression and metastasis (4, 5).

Quaking homolog (QKI) is part of the RNA-binding signal transduction and activation of RNA (STAR) protein family (6). The three major isoforms of QKI are QKI-5, QKI-6, and QKI-7 (7). QKI-6 binds to the QKI response element [QRE, NAUAAY-N(1–20)-UAAY] to regulate several physiological and pathological processes (6). QKI is a tumor suppressor in various human cancers, including oral, colon, gastrointestinal, and prostate cancers (8–11). Furthermore, QKI-5 could suppress lung cancer cell migration and invasion *via* EMT inhibition (12). QKI-5 is predominantly localized in the nuclear region, and QKI-6 can be localized in the nucleus and cytoplasm (13, 14). However, the role of QKI-6 in NSCLC remains unknown.

The protein disulfide isomerase (PDI) anterior gradient protein 2 (AGR2) is typically present in the endoplasmic reticulum (ER). AGR2 suppresses cancer cell proliferation and metastasis (15, 16). AGR2 is also considered a potential cancer biomarker in pancreatic, breast, lung, and colorectal cancers (17–20). However, there has been no report on the role and function of AGR2 and its link to QKI-6 in NSCLC to date.

Here, we aimed to determine the function of QKI-6 in NSCLC progression and elucidate the underlying mechanisms. Low QKI-6 expression was positively correlated with poor overall survival. Furthermore, QKI-6 inhibited cell proliferation, migration, and EMT processes by downregulating AGR2 in NSCLC. Our results provide novel insights into the role of QKI-6 in NSCLC, and QKI-6 may be used as a diagnostic and therapeutic target for NSCLC.

## Methodology

### Patients

NSCLC tissues and matched peritumoral tissues were collected from patients with NSCLC who underwent surgical resection in Tangdu Hospital affiliated with Air Force Military University (Xi’an, China). All the patients met the criteria of the WHO 2004 classification (21). Tumors were assigned stages according to the eighth TNM edition (22). Frozen NSCLC tissues and matched peritumor tissues were randomly selected for Western blot analysis. The study was approved by the Ethics Committee of Tangdu Hospital (Xi’an, China).

### Cell Lines and Cell Culture

Human lung cancer cell lines (A549) were purchased from the Type Culture Collection (Chinese Academy of Sciences, Shanghai, China). Cells were cultured in Dulbecco’s modified Eagle’s medium (DMEM, Gibco) supplemented with 10% fetal bovine serum (FBS, Gibco) and penicillin–streptomycin (Gibco) and incubated at 37°C with 5% CO_2_.

### Immunohistochemistry

The QKI expression pattern was studied using NSCLC tissue microarrays, which was purchased from Outdo Biotech Company (HLugA180su05, Shanghai, China). Immunohistochemistry (IHC) staining was performed using the QKI antibody (A7043, ABclonal) as previously described (23). IHC images were obtained using PANNORAMIC (3DHISTECH, Hungary), and the intensity of IHC staining was acquired using Image-Pro Plus 6.0 image software (Media Cybernetics, USA). The multiplied scores of IHC staining results were constructed based on intensity (0 = absent, 1 = faint yellow, 2 = reddish, 3 = brown) and percentage of stained positive cells (0 = 0%–5%, 1 = 6%–25%, 2 = 26%–50%, 3 = 51%–75%, 4 = 76%–100%). Scores of 0 to 5 were considered as low QKI expression, and scores of 6 to 12 were considered to indicate high QKI expression.

### Constructs and Establishment of Stable Cell Lines

Plasmids containing QKI-6 cDNA and plasmids expressing negative control shRNA (sh-NC) and QKI-6 shRNA (sh-QKI-6) were produced (QKI6-ShRNA-Forward: 5′-GATCCGCTGCTCCAAGGATCATTACTTTCAAGAGAAGTAATGATCCTTGGAGCAGCTTTTTTCTCGAGG-3′; QKI6-ShRNA-Reverse: 5′-AATTCCTCGAGAAAAAAGCTGCTCCAAGGATCATTACTTCTCTTGAAAGTAATGATCCTTGGAGCAGCG-3′), and the lentivirus was generated in 239T cells by Biowit Technologies (Shenzhen, China).

To obtain stable QKI-6 overexpression or knockdown, 1 × 10^5^ A549 cells were seeded per well in six-well plates (Corning) and grown to 75% confluence. After changing the culture medium, lentiviral particles containing QKI-6 cDNA, negative control, QKI-6 shRNA, or negative control shRNA were added. Cells were then cultured at 37°C with 5% CO_2_. The culture medium was removed and cells were isolated using 5 μg/ml puromycin for 24 h. Infection efficiency was evaluated by Western blot.

### Protein Extraction and Western Blotting

Total cellular protein from the A549 cell lines, NSCLC, and peritumoral tissues was extracted using RIPA buffer and quantified using the BCA assay. Sixty micrograms of protein from each sample was resolved by 10% SDS-PAGE, transferred to PVDF membranes (Millipore), which were then blocked in 5% skim milk in TBST, and incubated with primary antibody overnight at 4°C. The primary antibodies used were as follows: QKI (A7043, ABclonal), epidermal growth factor receptor (EGFR) (ab52894, ABclonal), AGR2 (1A8A8, Proteintech), E-cadherin (#14472, CST), N-cadherin (#13116, CST), p-SRC Y418 (ab40660, Abcam), and p-STAT3 Y705 (ab76315, Abcam). After washing with TBST, the membranes were incubated with horseradish peroxidase (HRP)-conjugated secondary antibodies for 2 h. Protein bands were visualized using a Tanon 5200 chemiluminescent system (Shanghai, China).

### CCK-8 Assay

Cell Counting Kit-8 (Beyotime, China, #c0037) was used to assess cell proliferation. A549 cells after gene transfection were plated in 96-well plates (1 × 10^3^ cells per well) for 1 to 5 days. The cells were treated with 20 μl sterile CCK-8 reagent per well and cultured for 4 h at 37°C. Absorbance was measured at 450 nm.

### Cell Proliferation Assay

A549 cell lines were plated in triplicate at a density of 800 cells in 60 mm dishes. On day 14, the colonies were stained with 0.1% crystal violet, and visible colonies were counted.

### Transwell Assays

A 24-well Transwell chamber was used for the migration assay. Cells (1 × 10^5^) were plated in the top chamber of an 8.0-μm membrane (Corning) Transwell in 200 μl DMEM without FBS. The bottom chamber contained 700 μl DMEM with 20% FBS. After 24 h, the migrated cells were fixed and stained with 0.1% crystal violet. Cells in six fields were counted randomly.

### Cell Cycle Analysis

A549 (5 × 10^5^) cells were plated in six-well plates and cultured for 20 h. After vortexing in PBS, the cell samples were fixed with 70% ethanol for 1 h. The cells were isolated by centrifugation and resuspended in PBS. The samples were incubated with propidium iodide (PI)/RNase (Thermo) for 30 min in the dark at room temperature and detected using a FACS Calibur (BD).

### Label-Free Quantification and LC/MS Proteomics Analysis

Sh-NC and sh-QKI-6 cells were cultured in 100 mm dishes. All the sample cells were treated using the Mhelix Biotech kit according to the manufacturer’s instructions (Shanghai, China). Label-free quantification and LC/MS proteomics analysis were performed by the Mhelix Biotech Company (Shanghai, China). The mass spectrometry proteomics data have been deposited to the ProteomeXchange Consortium *via* the PRIDE (24) partner repository with the dataset identifier PXD027013.

### qRT-PCR

Trizol reagent (Invitrogen) was used to isolate total RNA. According to the instruction of the M-MLV assay kit (Invitrogen), RNA was reverse-transcribed into cDNA. The primer sequences were as follows: AGR2-Forward: 5′-TTGTCCTCCTCAATCTGGTT-3′; AGR2-Reverse: 5′-ATCGGCTCTAACTGTCAGAGA-3′; GAPDH-Forward: 5′-GGGAAACTGTGGCGTGAT-3′; GAPDH-Reverse: 5′-GAGTGGGTGTCGCTGTTGA-3′. SYBR Green qPCR SuperMix for quantitative PCR was purchased from Vazyme Company. Quantitative PCR was performed using the ABI PRISM^®^ 7500 Sequence Detection System. Amplification conditions were set to 40 cycles at 95°C for 5 s and 65°C for 30 s.

### Statistics

We used GraphPad Prism version 8 (GraphPad software) for statistical analyses. Data were presented as mean ± standard deviation (SD). Student’s *t*-test, two-way ANOVA, or log-rank Cox was used for the statistical analyses. *P <*0.05 was considered statistically significant.

## Results

### QKI Downregulation Is Associated With Poor Prognosis in NSCLC

**Figure 1:**
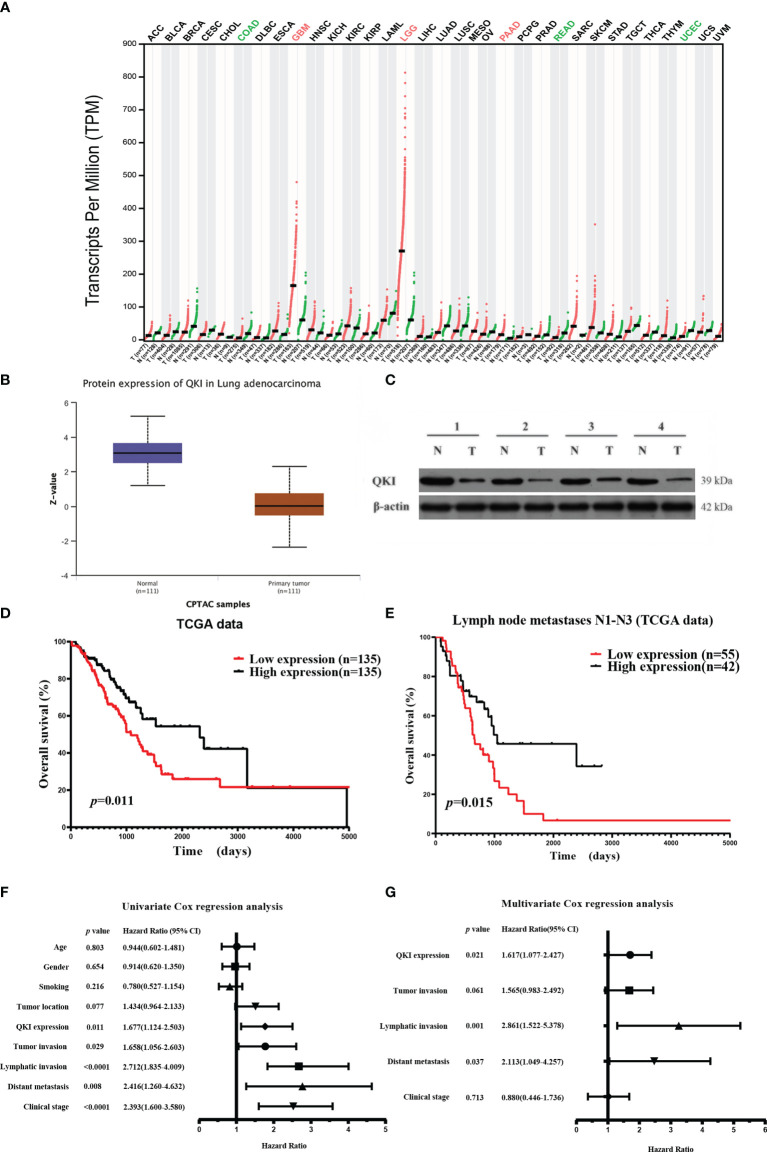
Elevated QKI expression improved prognosis in patients with non-small lung cancer (NSCLC). **(A)** QKI expression profile across several tumor samples and paired healthy tissues. **(B)** QKI protein expression levels in lung adenocarcinoma (NSCLC) and normal tissue samples were analyzed using CPTAC samples (*P* < 0.05). **(C)** QKI protein expression in tumor (T) and normal tissue (N) from four patients. **(D)** Kaplan–Meier (KM) overall survival (OS) analysis of QKI expression in lung adenocarcinoma by using the TCGA samples (*n* = 270; low expression vs. high expression; HR = 1.67, 95% CI = 1.13–2.47, *P* = 0.011, log-rank test). **(E)** KM OS analysis of QKI expression in patients with NSCLC with lymph node metastases N1–N3 in the TCGA samples (*n* = 97; low expression vs. high expression; HR = 1.93, 95% CI = 1.15–3.23, *P* = 0.015, log-rank test). **(F, G)** Univariate **(F)** and multivariate **(G)** analyses of the association between OS and clinical characteristics and QKI expression in the TCGA samples.

We first studied the putative functions of QKI-6 in NSCLC by comparing QKI mRNA expression in tumor and normal tissues. QKI-6 expression was lower in the NSCLC group than in the normal control group (GEPIA, Gene Expression Profiling Interactive Analysis, **Figure 1A**). Furthermore, QKI protein expression was higher in normal tissue than in NSCLC CPTAC (Clinical Proteomic Tumor Analysis Consortium) samples (*n* = 222, **Figure 1B**). QKI protein levels were significantly lower in tumor tissues than in normal tissues in four matched pairs of NSCLC and normal tissues (**Figure 1C**). Next, we analyzed the survival of 270 cases of NSCLC. Low QKI expression was positively correlated with worse NSCLC survival in the TCGA samples (HR = 1.677, 95% CI = 1.124–2.503, *P* = 0.011, **Figure 1D**). Kaplan–Meier analysis results based on 97 NSCLC cases with lymph node metastases N1–N3 showed that low QKI levels were correlated with poor prognosis (HR = 1.931, 95% CI = 1.154–3.232, *P* = 0.015, **Figure 1E**). Furthermore, Cox univariate analysis indicated that lymphatic invasion and distant metastasis were risk factors for overall survival in the TCGA samples (*P* < 0.05, **Figure 1F**). In the multivariate survival analysis, when other risk factors (*P* < 0.05, HR > 1 in univariate analysis) such as clinical stage, tumor invasion, lymphatic invasion, distant metastasis, and QKI expression were taken into the multivariate mode, QKI expression, lymphatic invasion, and distant metastasis had predictive value for OS in patients with NSCLC (**Figure 1G**).

### QKI Expression and Correlation With Clinical Results

**Figure 2:**
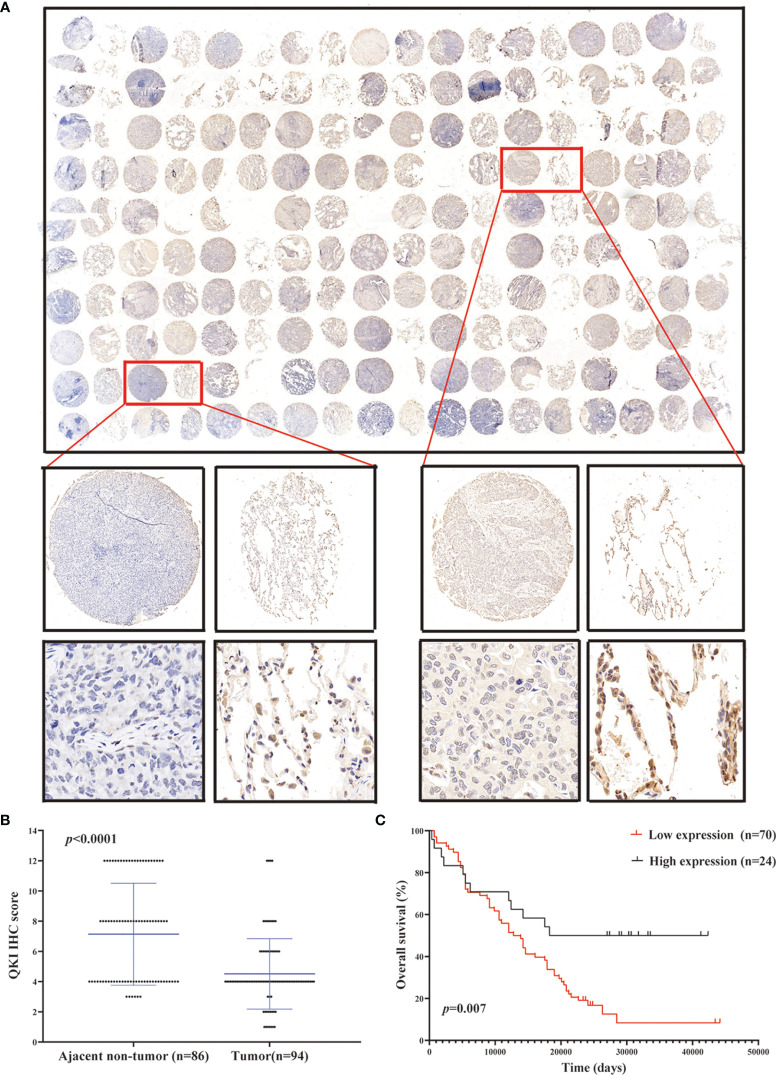
QKI downregulation in human NSCLC tissues. **(A)** Representative images of tumor and peritumor tissues after QKI staining. Scale bar, 200 and 50 μm (inset), respectively. **(B)** QKI expression was lower in tumor tissue than in ajacent non-tumor tissue (*P* < 0.0001). **(C)** KM plotter survival analysis based on microarray tissue IHC (immunohistochemistry) results containing 74 patients with NSCLC (low expression vs. high expression; HR = 2.24, 95% CI = 1.35–3.71, *P* = 0.007, log-rank test).

We performed IHC analysis to study the QKI level on the NSCLC tissue microarray containing 180 dots (86 pairs of tumor–normal tissues and 8 tumor dots, **Figure 2A**, **Table S1**). Among the tissue pairs, 54.7% (47/86) of the normal tissue samples and 25.5% (22/86) of the corresponding tumor tissue samples were identified as QKI positive. The QKI-6 expression levels in tumor tissues were significantly lower than those in normal tissues (staining scores: 4.51 vs. 7.14, *P* < 0.0001, **Figure 2B**). Moreover, downregulation of QKI expression in the NSCLC tissue microarray was correlated with poor prognosis, as seen in the results of the TCGA sample analysis (HR = 2.24; 95% CI: 1.35–3.71; *P* = 0.007, log-rank test, **Figure 2C**). When analyzing QKI expression with clinicopathological variables, we found that QKI level was significantly associated with NSCLC tumor invasion and clinical stage (**Table 1**).

**Table 1:** Association between QKI expression and clinical parameters in patients with NSCLC.

Clinical parameters	*n*	QKI expression		P-value
		Low	High	
Age				
>60	51	39	12	0.805
≤60	43	31	12	
Gender				
Male	51	40	11	0.091
Female	43	40	3	
Tumor invasion				
T1–T2	71	48	23	0.008*
T3–T4	23	22	1	
Lymph node metastases				
N0	57	43	14	0.979
N1–N3	37	27	10	
Clinical stage				
I	30	18	12	0.029*
II–IV	64	52	12	
Pathological stage				
Well and moderate	65	50	15	0.416
Poorly and not	29	20	9	
			

*Statistically significant (P < 0.05).

### QKI-6 Inhibits A549 Cell Proliferation and Migration

**Figure 3:**
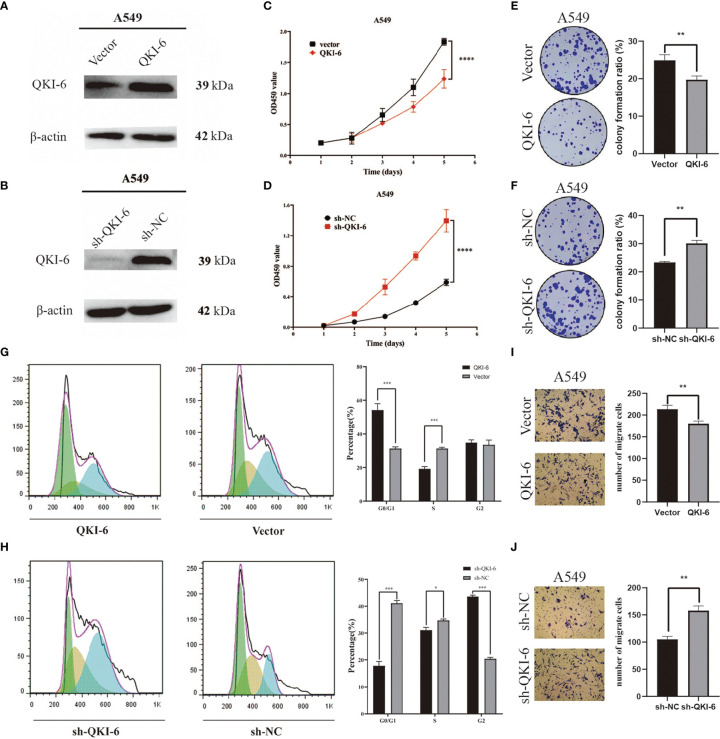
QKI-6 overexpression reduced proliferation and migration in NSCLC cells. **(A, B)** QKI-6 levels in QKI-6 overexpression A549 cell lines, QKI-6 shRNA interfering A549 cell lines, and normal A549 cell lines. **(C, D)** Growth curves of QKI-6 overexpression A549 cells, vector **(C)**, QKI-6 knockdown A549 cells, and sh-NC A549 cells **(D)**. Cell viabilities detected by the CCK-8 assay shown as optical density (OD) values. **(E, F)** Colony formation assay results showing the impact of QKI-6 knockdown and QKI-6 overexpression in A549 cells. **(G, H)** Flow cytometric study of the cell cycle. More cells overexpressing QKI-6 were arrested in the G0/1 phase, and more cells with QKI-6 knockdown entered into the S and G2 phases of the cell cycle. **(I, J)** Transwell migration assays using QKI-6 knockdown A549 cells and QKI-6 overexpression A549 cells. All assays were performed independently at least three times and data were presented as the mean ± SD; one-way ANOVA analysis of three independent experiments. *P < 0.05, **P < 0.01, ***P < 0.0001, ****P < 0.00001.

To determine whether QKI-6 could exert anticancer effects, QKI-6 overexpression and knockdown were performed in A549 cells (**Figures 3A, B** **)**. Overexpression of QKI-6 inhibited tumor cell proliferation, and knockdown of QKI-6 promoted cell proliferation (*P* < 0.0001, **Figures 3C, D** **)**. Furthermore, overexpression of QKI-6 significantly reduced cell colony formation ability (*P* < 0.01, **Figures 3E, F** **)** and induced a block in the G0/G1 phase. Conversely, knockdown of QKI-6 in A549 cells increased the proliferation and the proportion of cells in the S and G2 phases (**Figures 3G, H** **)**. Moreover, overexpression of QKI-6 significantly inhibited A549 cell migration, and knockdown of QKI-6 promoted A549 cell migration (**Figures 3I, J** **)**.

### Label-Free Quantitation of Differentially Expressed Proteins in sh-QKI-6 and sh-NC

**Figure 4:**
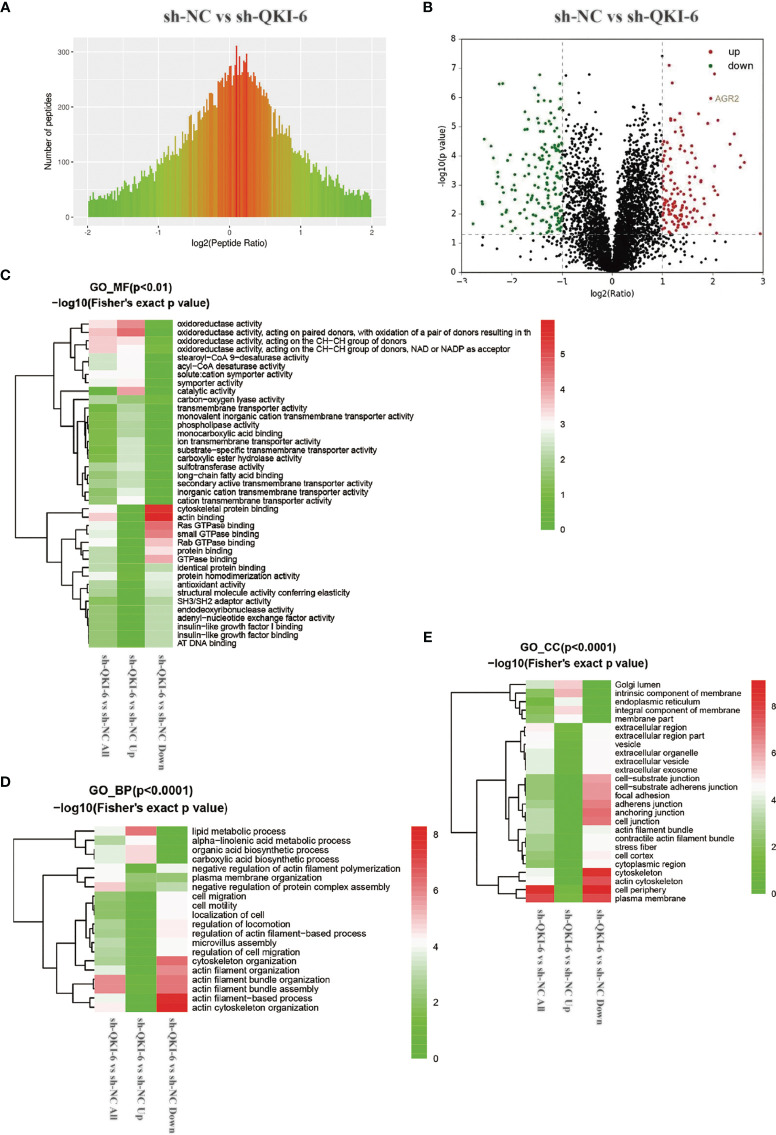
Different proteins and ontologies were identified by label-free quantitative proteomics technology. Functional annotation of dysregulated proteins analyzed by Protein Analysis Through Evolutionary Relationships (PANTHER) by using the Gene Ontology (GO) categories molecular function (MF), biological process (BP), and cellular component (CC). **(A)** Differences in peptide quantitative ratio distributions between knockdown QKI-6 A549 cells and control groups. **(B)** Volcano plot showing differential expression of proteins between knockdown QKI-6 A549 cells and control groups. **(C–E)** Heat map showing functional enrichment-based clustering for protein groups: GO molecular function cluster **(C)**, GO biological process function cluster **(D)**, and GO cellular component function cluster **(E)**.

To elucidate the mechanisms by which QKI-6 could promote A549 cell migration, label-free proteomics technology was used to detect changes in protein expression levels in sh-QKI-6 and sh-NC cells. We identified 4,373 differentially expressed proteins by using Proteome Discoverer 1.4 between the sh-QKI-6 and sh-NC cells (**Figure 4A**). The label-free quantitation analysis of sh-QKI-6 and sh-NC proteins was performed using the Progenesis LC-MS software to identify 316 differentially expressed proteins (>2-fold). Of these, 145 were upregulated and 171 were downregulated (sh-QKI-6/sh-NC >2-fold or <0.5-fold, *P* < 0.05, **Figure 4B**, **Table S1**). All the differentially expressed proteins were analyzed using the Gene Ontology (GO, www.geneontology.org) database, which provided networks of molecular function (MF, **Figure 4C**), biological process (BP, **Figure 4D**), and cellular component (CC, **Figure 4E**) to describe the gene product attributes (25). As shown in **Figures 4C–E**, the differentially expressed proteins were involved in various MFs and CCs. The classification of these proteins according to their molecular function showed that they were mainly involved in oxidoreductase activity, cytoskeletal protein binding, and actin binding (**Figure 4C**, **Figure S1**). We also found that QKI-6 played a significant role in BPs including lipid metabolism, cell migration, and cell motility (**Figure 4D**, **Figure S2**). The cellular component repartition analysis showed that the identified proteins were mainly presented in the cell periphery and plasma membrane (**Figure 4E**, **Figure S3**).

### QKI-6 Regulates Tumor-Related AGR2 and Inhibits EMT by Activating the EGFR–SRC–STAT3 Signaling Pathway

**Figure 5:**
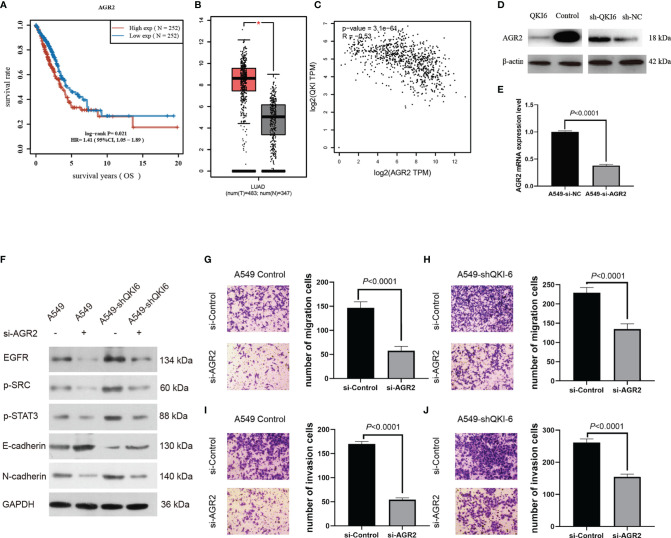
QKI-6 knockdown promotes NSCLC cell migration by inducing EMT *via* AGR2 upregulation. **(A)** Kaplan–Meier overall survival analysis of AGR2 expression in patients with NSCLC by using the TCGA samples (*n* = 504, *P* = 0.021, log-rank test). **(B)** AGR2 expression levels were analyzed in the NSCLC and normal control groups by using the TCGA and GTEx samples (red = tumor, *n* = 483; gray = normal, *n* = 347). **(C)** QKI expression was negatively correlated with that of AGR2 (*P* < 0.0001, *r* = −0.32, Spearman’s test). **(D)** Western blot analysis of AGR2 expression in QKI-6 overexpression A549 cell lines, QKI-6 shRNA A549 cell lines, and normal A549 cell lines. **(E)** AGR2 mRNA level was detected by qRT-PCR. **(F)** Effects of AGR2 knockdown in A549 and A549-shQKI-6 cells on p-SRC, p-STAT3, and EMT markers (E-cadherin, N-cadherin) detected by Western blot. GAPDH was used as an induced loading control. **(G, H)** Representative images and results of Transwell migration assays. **(I, J)** Representative images and results of Transwell invasion assays. Data in **(G–J)** are shown as the mean ± standard error from three independent experiments. *P < 0.05

Among the differentially expressed proteins, AGR2 was significantly upregulated in sh-QKI-6 A549 cells compared with sh-NC. We represented the result of AGR2 OS in the TCGA sample (*n* = 504) by Kaplan–Meier analysis. Higher AGR2 mRNA expression levels were correlated with poor prognosis (HR = 1.41, 95% CI = 1.05–1.89, *P* = 0.021, log-rank test, **Figure 5A**). Furthermore, in the TCGA and GTEx databases, AGR2 mRNA levels in NSCLC were significantly upregulated compared with those in normal tissues (*P* < 0.05, **Figure 5B**), and AGR2 mRNA levels were negatively correlated with QKI-6 mRNA levels (*n* = 513, *r* = −0.32, 95% CI = −0.39 to −0.23, *P* < 0.001, Spearman’s test, **Figure 5C**). In addition, Western blot analysis showed that AGR2 was significantly upregulated in sh-QKI-6 A549 cells (**Figure 5D**). In this study, AGR2 mRNA expression was analyzed in A549 cells to confirm the effectiveness of si-RNA-AGR2 (**Figure 5E**). EGFR, p-SRC, and p-STAT3 simultaneously increased in QKI-6 knockdown A549 cells and decreased in AGR2 knockdown A549 cells. QKI-6 expression also affected the expression of E-cadherin and N-cadherin (**Figure 5F**). Furthermore, siRNA-mediated AGR2 knockdown significantly decreased migration and invasion in control and QKI-6 knockdown A549 cells (**Figures 5G–J**, *P* < 0.01). Thus, QKI-6 is likely an inhibitor of NSCLC metastasis and suppresses EMT and metastasis by regulating AGR2 *via* EGFR–SRC–STAT3 signaling.

## Discussion

QKI-6 is a tumor-suppressive regulator in various human cancers including bladder cancer (26), pancreatic cancer (27), clear cell renal cell carcinoma (28), and glioblastoma (29). QKI-5 could reduce the progression of lung cancer (12). It was reported that QKI-6 might affect colorectal cancer through β-catenin and p27^Kip1^ signaling (30). Furthermore, QKI-6 could downregulate E2F3 and NF-κB signaling to inhibit bladder cancer malignant behaviors (26). However, whether QKI-6 could inhibit NSCLC cell migration and the underlying mechanism was unclear. We showed that QKI-6 played a similar role in NSCLC; QKI-6 expression was downregulated in NSCLC, which resulted in EMT by upregulating AGR2 expression in NSCLC. These data indicated that QKI-6 could be developed as a therapeutic target in patients with NSCLC.

To determine the effects of QKI-6 in NSCLC, we overexpressed or knocked down QKI-6 in A549 cells and confirmed that QKI-6 mediated a reduction in NSCLC cell proliferation. Furthermore, QKI-6 overexpression blocked the cells at the G0/G1phase, while QKI-6 knockdown promoted tumor cell arrest at the S and G2/M phases. Our data reiterated the anticancer role of QKI-6, and our study is the first to date to show the role of QKI-6 as a tumor-suppressor gene in NSCLC.

To elucidate the molecular mechanism by which QKI-6 induces NSCLC cell migration, we first performed label-free quantification (LFQ) to identify genes that are differentially expressed in sh-QKI-6 and sh-NC. The LFQ proteomics approach improved our understanding and provided clearer insights into the QKI-6-mediated molecular changes and putative mechanisms and potential diagnostic biomarkers (31, 32). In the present study, 4,373 proteins were quantified, and 145 upregulated proteins and 171 downregulated proteins were differentially expressed (fold >2 or <0.5, *P* < 0.05). However, the low precision and inaccuracy of LFQ was a technical challenge. We tested the novel gene AGR2 involved in the positive regulation of the EGFR signaling pathway, which played an important role in the metastasis of NSCLC (sh-QKI-6 vs. sh-NC = 3.902, *P* < 0.0001). AGR2 is a potential oncogenic biomarker, which is overexpressed in many patients with NSCLC with short survival duration (33–35). AGR2 could regulate EGFR expression at the plasma membrane. Because of an overlap in cellular effects, the major effects of AGR2 are regulated *via* EGFR signaling (36, 37). However, EGFR expression was not involved in AGR2-dependent oncogenic processes (38). In the present study, we showed that EGFR and AGR2 expression were upregulated in sh-QKI-6 A549 cells.

EGFR–SRC–STAT3 signaling plays a vital role in some human cancers. Dosch et al. reported that activation of EGFR–SRC–STAT3 signaling induced stromal remodeling and improved pancreatic cancer cell survival (39). Co-targeting STAT3 and EGFR or STAT3 and SRC suppressed cell growth in pancreatic cancer (40). Li et al. reported that EGFR–SRC–STAT3 signaling played a significant role in conferring resistance to sorafenib in hepatocellular carcinoma (41). In our study, we observed a marked change in SRC, STAT3, and EMT marker (E-cadherin and N-cadherin) expression. However, the mechanism underlying this signaling in NSCLC progression requires further investigation. In summary, our results indicated that QKI-6 played an essential role in regulating EGFR-mediated EMT signaling by regulating AGR2 expression.

Taken together, our results showed that QKI-6 could negatively regulate NSCLC cell proliferation, migration, and cell cycle progression. Furthermore, QKI-6 overexpression could downregulate AGR2 expression and inhibit the EMT process. Therefore, targeting this pathway could be a potential therapeutic strategy in patients with NSCLC.

## Data Availability Statement

The datasets presented in this study can be found in online repositories. The names of the repository/repositories and accession number(s) can be found below: http://proteomecentral.proteomexchange.org/cgi/GetDataset, PXD027013.

## Ethics Statement

The studies involving human participants were reviewed and approved by the Ethics Committee of Tangdu Hospital. The patients/participants provided their written informed consent to participate in this study. Written informed consent was obtained from the individual(s) for the publication of any potentially identifiable images or data included in this article.

## Author Contributions

Conceptualization: YN and TZ. Methodology: XS. Software: YN. Validation: HZ, JL, and XW. Formal analysis: LZ. Resources: DT. Data curation: LZ. Writing—original draft preparation: HZ. Writing—review and editing: FT and TZ. Visualization: YN. Supervision: FT and YN. Project administration and funding acquisition: YN. All authors have read and agreed to the published version of the manuscript.

## Funding

This work was supported by a grant from the National Natural Science Foundation of China (grant number 81301989 to YN).

## Conflict of Interest

The authors declare that the research was conducted in the absence of any commercial or financial relationships that could be construed as a potential conflict of interest.

## Publisher’s Note

All claims expressed in this article are solely those of the authors and do not necessarily represent those of their affiliated organizations, or those of the publisher, the editors and the reviewers. Any product that may be evaluated in this article, or claim that may be made by its manufacturer, is not guaranteed or endorsed by the publisher.
