# Supplementary figures and images for: QKI-6 Suppresses Cell Proliferation, Migration, and EMT in Non-Small Cell Lung Cancer
Source: Front Oncol. 2022 May 5;12:897553. doi: 10.3389/fonc.2022.897553 (PMC9117621; doi:10.3389/fonc.2022.897553)

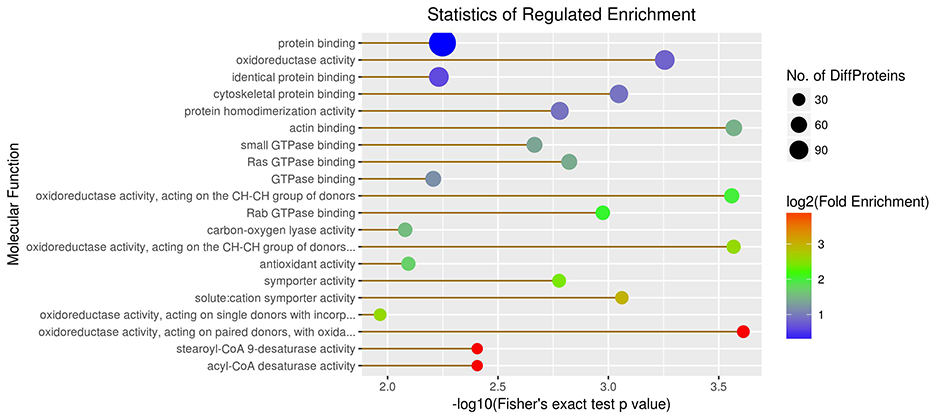

Supplement: Supplementary file 1 [file Image_1.tif]

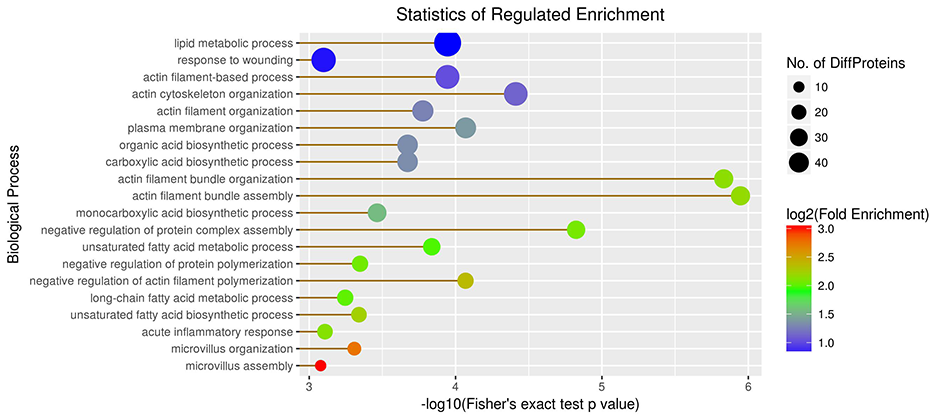

Supplement: Supplementary file 2 [file Image_2.tif]

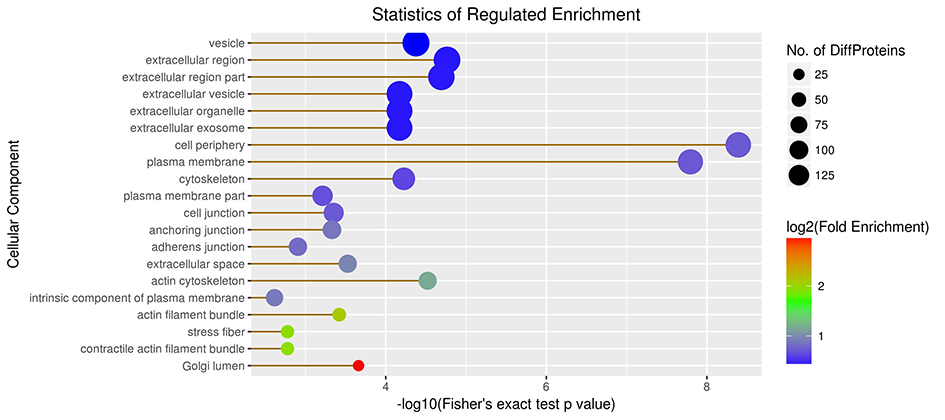

Supplement: Supplementary file 3 [file Image_3.tif]
